# Penalized Cumulative Probability Model for a Continuous Outcome Subject to Detection Limits

**DOI:** 10.1002/sim.70723

**Published:** 2026-08-28

**Authors:** Shuai Sun, Valeria R. Mas, Kellie J. Archer

**Affiliations:** ^1^ Division of Biostatistics College of Public Health, The Ohio State University Columbus Ohio USA; ^2^ Surgical Sciences Division, Department of Surgery University of Maryland School of Medicine College Park Maryland USA

**Keywords:** false discovery rate, generalized monotone incremental forward stagewise, ordinal regression, regularized model

## Abstract

Mixed‐type outcome data occur when the outcome variable's distribution is a mixture of both continuous and discrete ordinal variables. Such mixed‐type outcomes are common in biomedical, psychological, and the health sciences, particularly for variables having either a detection or quantitation limit. When interest lies in identifying a combination of genomic features associated with a mixed‐type outcome, any method used would require a variable selection strategy for high‐dimensional data. Unfortunately, few variable selection methods exist for modeling a mixed‐type outcome when the covariate space is high dimensional. This study develops a high‐dimensional penalized cumulative probability model (CPM), to allow for the identification of genomic features associated with mixed‐type outcome of interest. We demonstrated how such model may be estimated using the iterative penalization procedure—the generalized monotone incremental forward stagewise (GMIFS) algorithm. The Model‐X knockoffs procedure was combined with the estimation algorithm to control the false discovery rates (FDR) when performing variable selection. Through extensive simulation studies, our penalized CPM was shown to outperform alternative methods in terms of controlled variable selection performance by achieving high statistical power with the FDR being controlled at the target level. We demonstrate the utility of our method by applying it to predict estimated glomeruli filtration rate (eGFR) in kidney transplant recipients at 24 months post‐transplant using baseline gene expression data as predictors. Our CPM model identified five genes associated with this mixed‐type outcome which have important links to renal disease, which may provide prognostic guidance for kidney transplantation recipients.

**Abbreviations**
DL: detection limit
eGFR: glomerular filtration rate
FDR: false discovery rate
GMIFS: generalized monotone incremental forward stagewise
LOD: limit of detection

## 1 Introduction

In biomedical, psychological, and health sciences, studies frequently encounter outcomes of a “mixed type,” where the response variable distribution is a mixture of both continuous observations and discrete ordinal categories. Such mixed‐type outcomes typically arise due to a detection limit (DL) or a quantitation limit [1]. A premier motivating example of this statistical challenge is the estimation of glomerular filtration rate (eGFR) used to monitor kidney transplant recipients post‐transplant [2, 3]. When using eGFR as a proxy for kidney function, values below $60\;\mathrm{mL}/\min/1.73m^{2}$ are reported as precise continuous measurements, whereas values ≥60 lack clinical precision and are universally right‐censored and reported as a single coarsened category (“$\geq 60^{\text{”}}$) [3, 4]. Analyzing such data via arbitrary dichotomization (e.g., thresholding graft function at 45) discards substantial numeric information, severely undermining statistical power and inferential efficiency [2]. Preserving the full distributional framework requires methods capable of modeling the mixed‐type outcome directly.

More generally, left‐censoring at a limit of detection (LOD) yields an orderable categorical level for non‐detects alongside continuous values for remaining positive signals [1, 5]. Similarly, right‐censoring at a limit of quantification generates bounded thresholds where values beyond the limit are coarsened. Various ad‐hoc substitution procedures have historically been evaluated to manage data subject to an LOD, such as omitting values [6], setting them to zero [6, 7], substituting simple fractions like LOD/2 [8] or $\mathrm{LOD}/\sqrt{2}$ [6], or applying maximum likelihood extrapolations [9, 10]. However, these approaches routinely introduce severe estimation bias, compress sample variance, or fail entirely when a substantial proportion of the data falls outside the quantifiable range [6]. More rigorous recent alternatives include Bayesian imputation frameworks under censored Gaussian assumptions [7]. Yet, robust models capable of directly accommodating the mixed continuous‐ordinal architecture of these response variables remain scarce.

A promising avenue for resolving this challenge is the cumulative probability model (CPM) studied by Liu et al. [11]. Taking advantage of the mathematical equivalence between CPMs and semiparametric linear transformation models [12], the CPM can simultaneously map continuous response variables, discrete ordinal metrics, and mixed‐type structures by defining an appropriate intercept function [11]. Crucially, however, existing developments of the CPM are restricted to low‐dimensional configurations where the number of potential predictors (P) is smaller than the sample size (N).

In modern translational medicine, there is a critical need to utilize high‐throughput genomic data—such as deceased donor organ gene expression profiles—to accurately predict clinical outcomes like post‐transplant eGFR [2, 13]. While penalized ordinal regression algorithms, such as those utilizing the generalized monotone incremental forward stagewise (GMIFS) algorithm [14], execute effective variable selection in high‐dimensional settings [15], they are strictly limited to discrete ordinal responses with a few levels (e.g., 3 to 5 groups) and fail when confronted with mixed‐type outcomes or responses featuring extensive numeric gradations [16, 17]. A statistical method gap thus persists between high‐dimensional regularized models and the flexible continuous‐ordinal CPM framework.

To bridge this gap, this study develops a high‐dimensional penalized CPM specifically optimized for a continuous outcome subject to detection or clinical reporting limits (i.e., a mixed‐type outcome) as well as for non‐linear continuous outcomes. We implement an estimation path utilizing the regularized GMIFS algorithm [14] to screen high‐throughput genomic predictors, combined with the Model‐X knockoffs framework [18] to guarantee strict control of the false discovery rate (FDR). The remainder of this article is organized as follows: Section 2 outlines the statistical framework, likelihood formulations, and the knockoff selection algorithm. Section 3 details comprehensive simulation studies evaluating variable selection performance under various settings and link functions. Section 4 applies our method to identify prognostic baseline gene expression biomarkers predictive of 24‐month mixed‐type eGFR in kidney transplantation recipients. While eGFR ≥60 represents a clinical reporting threshold arising from estimation imprecision rather than a true assay‐driven analytical limit of quantification (LOQ), the resulting coarsened data structure represents an analogous statistical challenge that serves as a useful illustrative application for our proposed GMIFS CPM framework. Section 5 concludes with a discussion.

## 2 Statistical Models and Algorithms

### 2.1 CPM

Ordinal response models are generally proposed to model ordinal categorical response variables, where the most common class of ordinal regression models is the ordinal CPM systemically studied by McCullagh [19]. The general form of an ordinal CPM for a discrete ordinal response Y taking one of K ordinal levels (j=1,2,…,K−1) is written as $\text{link}\left\{P\left[Y\leq j|X\right]\right\}=\text{link}\left\{\gamma_{j}\left(X\right)\right\}=\theta_{j}-\beta^{T}X$, where “${\text{link}}^{\text{”}}$ represents a pre‐specified link function and elements in θ are “${\text{cutpoints}}^{\text{”}}$ on the link function transformed scale, but generally of little interest [19]. The regression coefficients β capture the stochastic association between the ordinal outcome and covariates and parameter estimation of ordinal CPMs can be obtained by the maximum likelihood estimation (MLE) method [19].

The motivation of applying ordinal CPMs to continuous outcomes is simple and intuitive: continuous data are also ordinal and thus ordinal regression models can be fit to continuous outcomes [11]. The methodological rationale supports this idea based on the equivalence between ordinal CPMs and another approach called the linear transformation model [11, 12]. In a linear transformation model, the observed outcome Y is modeled as a monotonic transformation of a latent variable $Y^{*}$, which is assumed to be modeled linearly with some specified error distribution [11, 12]. Then, the general form of the linear transformation model is defined as follows:

(1) $$Y=H\left(Y^{*}\right)=H\left(\beta^{T}X+\epsilon \right)$$

where H(·) is an increasing but unknown transformation function and ϵ represents random error with a specified error distribution, $F_{\epsilon }$. Based on the model in [1], we can derive the conditional cumulative probability function of observed outcome Y given covariates X such that,

$$\begin{array}{cc} F\left(y|X\right) & =P\left(Y\leq y|X\right)\\ & =P\left[H\left(\beta^{T}X+\epsilon \right)\leq y|X\right]\\ & =P\left[\epsilon \leq H^{-1}\left(y\right)-\beta^{T}X|X\right]\\ & =P\left[\epsilon \leq H^{-1}\left(y\right)-\beta^{T}X\right]\\ & =F_{\epsilon }\left[H^{-1}\left(y\right)-\beta^{T}X\right]. \end{array}$$

with some re‐formatting of the parameters by defining $G=F_{\epsilon }^{-1}$ and $\alpha =H^{-1}$, where G(·) is the link function and α(·) is the intercept function, the associated CPM for continuous response variables proposed by Liu et al. [11] is,

(2) $$G\left[P\left(Y\leq y|X\right)\right]=\alpha \left(y\right)-\beta^{T}X.$$

Therefore, the linear transformation model (Equation 1) is equivalent to the CPM (Equation 2).

Taking advantage of the equivalence between the CPM (Equation 2) and the linear transformation model (Equation 1), because different types of transformation functions H(·) determine the type of observed outcome Y we actually model in a linear transformation model, the associated CPM [5] can model other types of ordered outcome data besides discrete ordinal data. For example, if H(·) is a continuous function, the observed response variable Y can be a continuous variable. If H(·) is constant on some intervals but continuous elsewhere, the observed Y can be a mixed type of continuous and discrete ordinal variables. In the extreme case where H(·) is a left‐continuous nondecreasing step function, the observed outcome Y is a discrete ordinal variable modeled by traditionally defined ordinal CPMs. Therefore, the CPM (Equation 2) can be applied to different types of orderable outcome data, including discrete ordinal, continuous, and mixed type of discrete ordinal and continuous variables [11].

Denote the observed outcome of interest as Y with realized observations $\left\{y_{i};i=1,\ldots ,n\right\}$ with K ordered distinct values (i.e., ordinal levels). For a continuous response, without loss of generality we assume each observed value is unique (i.e., without ties), representing its own ordinal level and is always ordered in the manner where $y_{1}\leq \ldots \leq y_{n}$. Then the observed value $y_{i}$ of Y is equivalent to its ordinal level with the number of ordinal levels equal to the number of observations (i.e., sample size). On the other hand, suppose outcome Y is a mixed‐type response variable with either a LOD τ and/or a limit of quantification ψ, where values of Y below the LOD or above the limit of quantification are considered unreliable to report and analyze [1] and thus truncated or censored at some constants, which further consists of undetectable categories. In such a case, the number of ordinal levels is less than the sample size. Additionally, for high‐dimensional covariate spaces we need a solution when the number of covariates (P) exceeds the number of samples (n). Thus, our proposed CPM for a continuous outcome subject to DLs (i.e., mixed‐type outcome) is defined as follows:

(3) $$G\left[P\left(Y\leq y_{\left(i\right)}|x_{i}\right)\right]=\alpha \left(y_{\left(i\right)}\right)-\beta^{T}x_{i}=\alpha_{y_{\left(i\right)}}-\beta^{T}x_{i},$$

where $y_{\left(i\right)}$ represents the ordinal level that the ith subject's observed value $y_{i}$ belongs to, which takes on integer value (i.e., $y_{\left(i\right)}=1,2,\ldots ,K$), X is the standardized penalized covariate matrix consisting of P penalized variables, and $x_{i}$ corresponds to the observed values of covariates vector for ith subject. For parameter estimation, α and β are two sets of parameters, where the $\alpha_{k}$'s are the intercepts of little interest and β represents the regression coefficients for the penalized covariates with P dimensions, which are our primary interest. Subsequently, the approximate nonparametric likelihood function for our CPM (Equation 3) is given by,

(4) $$\begin{array}{c} L^{*}\left(\beta ,\alpha \right)=\prod_{i=1}^{n}\pi_{i}=\prod_{i=1}^{n}\left[G^{-1}\left(\alpha_{y_{\left(i\right)}}-\beta^{T}x_{i}\right)-G^{-1}\left(\alpha_{y_{\left(i\right)}-1}-\beta^{T}x_{i}\right)\right]\\ =\prod_{i=1}^{n}\left[F_{\epsilon }\left(\alpha_{y_{\left(i\right)}}-\beta^{T}x_{i}\right)-F_{\epsilon }\left(\alpha_{y_{\left(i\right)}-1}-\beta^{T}x_{i}\right)\right], \end{array}$$

where $\pi_{i}$ represents the probability for each subject {i=1,…,n}, $G^{-1}=F_{\epsilon }$ is the inverse of link function and $F_{\epsilon }$ is the specified error cumulative distribution function (CDF). Without loss of generality, we always assume $y_{1}\leq \ldots \leq y_{n}$ (i.e., the data are ordered in ascending order of their observed values of the outcome Y). In light of the derivation of the nonparametric MLE for the ordinal CPM (Equation 2) by Liu et al. [11], we also introduce an auxiliary parameter $\alpha_{0}\left(<\alpha_{1}\right)$ and always assume ${\hat{\alpha }}_{0}=-\infty$ and ${\hat{\alpha }}_{K}=+\infty$ corresponding to the largest ordinal level K. Then, the corresponding nonparametric log‐likelihood function is written as,

(5) $$\begin{array}{cc} l^{*}\left(\beta ,\alpha \right) & =\log L^{*}\left(\beta ,\alpha \right)\\ & =\sum_{i=1}^{n}\log\left(\pi_{i}\right)\\ & =\sum_{i=1}^{n}\log\left[F_{\epsilon }\left(\alpha_{y_{\left(i\right)}}-\beta^{T}x_{i}\right)-F_{\epsilon }\left(\alpha_{y_{\left(i\right)}-1}-\beta^{T}x_{i}\right)\right]. \end{array}$$

### 2.2 Generalized Monotone Incremental Forward Stagewise

Penalized regression methods such as least absolute shrinkage and selection operator (LASSO) [20], ridge regression [21, 22], elastic net [23] and least angle regression (LAR) [24] have been demonstrated to show excellent performance when applied to high‐throughput genomic data sets. They can prevent overfitting by providing penalized coefficient estimates for all covariates and thus trade unbiasedness for smaller variance and improved prediction accuracy. In the linear regression setting, the incremental forward stagewise (IFS) method is a version of boosting which produces a strikingly similar coefficient profile to the $L_{1}$‐penalized regression (LASSO) profile [25]. The IFS algorithm is a greedy procedure similar to the commonly used forward stepwise regression method in linear models but is more cautious, where the IFS works by incrementing the coefficient of the variable most correlated with the current residuals by an amount ±ϵ at each iteration step. When the incremental amount ϵ↓0, the IFS algorithm (called ${\mathrm{FS}}_{0}$) produces an identical solution path to the LASSO path under certain conditions [24]. ${\mathrm{FS}}_{0}$ can be characterized as a monotone version of the LASSO with much smoother regularization paths [14]. However, it is inappropriate to find the candidate covariate for updating based on the correlation with the residuals for non‐linear models. Alternatively, a generalization of this characterization to problems involving other than squared error loss, such as the logistic regression model, has been proposed and referred to as the generalized monotone incremental forward stagewise (GMIFS) method [14]. At each step, the GMIFS algorithm identifies the covariate having the largest gradient of the model log‐likelihood evaluated at the current parameters estimates and updates the corresponding coefficient by a small incremental amount. Therefore, the GMIFS can be applied to any statistical model for which the gradient function can be derived, and it has been proven useful in a wide variety of high‐dimensional settings for modeling different outcomes, including ordinal [15, 26], discrete survival time [27], count responses [28], and time‐to‐event models [29].

We extended the CPM to handle both continuous and mixed‐type outcomes in a high‐dimensional covariate space, via the GMIFS algorithm. Following the same notation used in Section 2.1, our GMIFS CPM for either continuous or mixed‐type outcome where the covariate space is high‐dimensional (i.e., high‐throughput genomic features) is written as follows:

(6) $$G\left[P\left(Y\leq y_{\left(i\right)}|{\tilde{x}}_{i}\right)\right]=\alpha \left(y_{\left(i\right)}\right)-\beta^{T}{\tilde{x}}_{i}=\alpha_{y_{\left(i\right)}}-\beta^{T}{\tilde{x}}_{i}$$

where $y_{\left(i\right)}$ represents the ordinal level as defined for model (Equation 3) in Section 2.1, whereas $\tilde{X}=\left[X,-X\right]$ is the augmented standardized covariate matrix consisting of P variables (X) which is augmented by appending −X to reduce the computational burden of determining the direction of update (i.e., the sign of coefficient) via second derivatives. Therefore, the corresponding penalized covariate coefficients β are $\beta =\left(\beta^{+},\beta^{-}\right)$ with 2P dimensions. The nonparametric likelihood and log‐likelihood functions of GMIFS CPM in Equation (6) share the same formulations as shown in Equation (4) and Equation (5), respectively, with the exception that we replace the original standardized covariate matrix consisting of P variables, X with the augmented standardized covariate matrix consisting of P variables and their corresponding P negative versions, $\tilde{X}$. Finally, the gradient function of the model log‐likelihood with respect to $\beta_{k}$ (k=1,2,…,2P) for GMIFS CPM (Equation 6) can be derived in the following form:

(7) $$\begin{array}{cc} \frac{\partial \log L^{*}}{\partial \beta_{k}} & =\frac{\partial l^{*}}{\partial \beta_{k}}=\frac{\partial }{\partial \beta_{k}}\sum_{i=1}^{n}\log\left[F_{\epsilon }\left(\alpha_{y_{\left(i\right)}}-\beta^{T}{\tilde{x}}_{i}\right)-F_{\epsilon }\left(\alpha_{y_{\left(i\right)}-1}-\beta^{T}{\tilde{x}}_{i}\right)\right]\\ & =\sum_{i=1}^{n}\left(-{\tilde{x}}_{\mathrm{ik}}\right)\frac{F_{\epsilon }^{\prime }\left(\alpha_{y_{\left(i\right)}}-\beta^{T}{\tilde{x}}_{i}\right)-F_{\epsilon }^{\prime }\left(\alpha_{y_{\left(i\right)}-1}-\beta^{T}{\tilde{x}}_{i}\right)}{F_{\epsilon }\left(\alpha_{y_{\left(i\right)}}-\beta^{T}{\tilde{x}}_{i}\right)-F_{\epsilon }\left(\alpha_{y_{\left(i\right)}-1}-\beta^{T}{\tilde{x}}_{i}\right)}, \end{array}$$

where $F_{\epsilon }^{\prime }$ denotes the first‐order derivative function of $F_{\epsilon }$ and ${\tilde{x}}_{\mathrm{ik}}$ represents kth covariate for the ith subject.

Our GMIFS algorithm proceeds in an iterative fashion where it updates one of the penalized coefficients $\beta_{k}$ among $\left(\beta^{+},\beta^{-}\right)$ associated with the largest gradient by a small incremental amount at each iteration step. Specifically, the selected penalized coefficients were updated with a trivial incremental amount ϵ (set to be 0.001 by default) so that the solution paths for both positive and negative coefficients are constrained to be monotonically nondecreasing at each iteration. At the end of the iterative procedure, we obtained the final solution path for the original penalized coefficients β with P dimensions of our interest by subtracting the coefficient estimates for the negative parts of the variables from those for the positive counterparts. The GMIFS algorithm for the penalized CPM for both continuous and mixed‐type outcomes with high‐dimensional covariate space is summarized in Algorithm 1. The algorithm starts by standardizing the penalized covariates X and then augmenting them by their corresponding negative values to create an expanded penalized design matrix, $\tilde{X}=\left[X,-X\right]$. Then the expanded penalized covariate coefficients β are initialized as $\beta_{1}=\beta_{2}=\ldots =\beta_{P}=\beta_{P+1}=\ldots =\beta_{2P}=0$ (i.e., $\beta^{+}=\beta^{-}=\vec{0}$). The nuisance parameters α are then initialized via nonparametric MLE based on the null model (i.e., intercept‐only model) given $\beta =\vec{0}$. To update the penalized parameters, the algorithm finds the candidate covariate based on the largest gradient and updates its coefficient by a small incremental amount given current parameter estimates. The algorithm proceeds by updating the model log‐likelihood and corresponding gradients using the most recent penalized parameter estimates. Therefore, our GMIFS algorithm iterates between updating the penalized parameters and updating gradients until some stopping criterion is met. The stopping criterion was set to when the difference between two successive log‐likelihoods falls below a pre‐specified tolerance τ (set to $10^{-4}$). At that point, the iterative procedure is considered to have converged and the algorithm stops. To prevent over‐fitting, we used the modified AIC (mAIC), a modified extension of traditional AIC specifically designed for high‐dimensional covariate space settings [30, 31, 32], to select the optimal step for producing the final estimates. Our algorithm additionally provides the modified BIC (mBIC) criterion for users interested in that information criterion. The exact forms of mAIC and mBIC were defined as [32],

$$\begin{array}{ll} \text{mAIC} & =2k_{M}+2\log\left(2P\right)k_{M}-2\log\left(k_{M}!\right)-2\text{loglikelihood},\\ \text{mBIC} & =k_{M}\log\left(n\right)+2\log\left(P/4\right)k_{M}-2\log\left(k_{M}!\right)-2\text{loglikelihood}, \end{array}$$

where $k_{M}$ represents the model size (i.e., number of nonzero regression coefficients in the model), P represents the total number of predictors, and n is the sample size. Again, we determined the optimal step of our GMIFS CPM for producing the final estimates as that attaining the minimum mAIC. Note that we always fix the intercepts α at initialized values instead of updating them after fixing $\beta^{\left(s+1\right)}$ at each iteration. The main reasons for this include: (1) the intercept parameters α are essentially nuisance parameters and are generally of little to no interest [19]; (2) the intercept parameters are usually referred to as “cutpoints” for discrete ordinal levels on the link function transformed scale, however, when the outcome of interest is a continuous response without ties, these “cutpoints” are not that important; (3) the parameter α can also be interpreted as the intercept function α(·), which represents the best back‐transformation needed for observed outcome Y such that $Y^{*}=\alpha \left(Y\right)$ can be fit with a linear regression model with error term $\epsilon ∼F_{\epsilon }\left(=G^{-1}\right)$ [11]. However, our proposed penalized method focuses on identifying important features and utilizing these features to make predictions of non‐linear continuous or mixed‐type outcomes as precise as possible whereas estimation of such best back‐transformation needed for outcomes is not our interest. Therefore, as long as estimates of these intercepts α can preserve the order information of response variables, we do not need to update them iteratively.

ALGORITHM 1 **GMIFS algorithm for penalized cumulative probability models.**

1. Standardize all P penalized predictors X and augment the covariate space by their corresponding negative values to prepare the expanded design matrix $\tilde{X}=\left[X,-X\right]$.
2. Initialize β at step s=0 as $\beta^{+}=\beta^{-}=\vec{0}$.
3. Extract the levels as distinct ordered values from outcome Y and match each observed value $y_{i}$ to its corresponding ordinal level $y_{\left(i\right)}$ for subject i, where i=1,…,n.
4. Initialize the intercepts α via nonparametric MLE.
5. Considering α fixed, find the predictor $m=\arg\max_{k}\frac{\partial l^{*}}{\partial \beta_{k}}$ for k=1,2,…,2P, and update $\beta_{m}^{\left(s+1\right)}\leftarrow \;\beta_{m}^{\left(s\right)}+\epsilon$.
6. Update the model log‐likelihood and corresponding gradients based on the most current estimate $\beta^{\left(s+1\right)}$.
7. Repeat steps 5 and 6 until $\log L^{\left(s+1\right)}-\log L^{\left(s\right)}<\tau$ where τ is pre‐specified tolerance.

Furthermore, we combined our proposed GMIFS CPM with the Model‐X knockoffs approach to allow for strict control of the FDR. In the Model‐X knockoffs framework, a set of so‐called “knockoff” random variables which are independent of the response conditionally on the original covariates, but whose structure mirrors that of the original covariates, are constructed [18]. These knockoff variables can then be used as negative controls for the original covariates, so that only original covariates that are considerably more associated with the response relative to their corresponding knockoff are selected, thus teasing apart true signals from noise [18]. In practice, the Model‐X knockoffs procedure can be implemented in four steps:

1. Construct Model‐X knockoff random variables for the original covariates, $\tilde{X}$.
2. Calculate the knockoff feature statistics, W ($W_{j}:j\in \left\{1,\ldots ,P\right\}$), based on the extended design matrix, $\left[X,\tilde{X}\right]$ and the response Y.
3. Determine the data‐dependent rejection threshold, τ.
4. Select important features as those achieving $W_{j}\geq \tau$

Knockoffs can be generated under a multivariate Gaussian assumption of the joint distribution of $\left(X,\tilde{X}\right)$ that $\left(X,\tilde{X}\right)∼N\left(0,G\right)$ if one believes that their predictors X are Gaussian [18]. Because we did not want to make distributional assumptions, we generated Model‐X knockoffs using the second‐order moment approximation method [18], which requires that ${\left(X,\tilde{X}\right)}_{\text{swap}\left(S\right)}$ and $\left(X,\tilde{X}\right)$ have the same first two moments (i.e., the same mean and covariance) rather than requiring them to have the same distribution for any subset S (i.e., exchangeability of the original and knockoff variables under swapping) [18]. This was accomplished using the approximate semidefinite program (ASDP) construction procedure to approximate the covariance matrix, which should be positive semidefinite as previously indicated [18]. We note that the fundamental theoretical requirement for valid FDR control in the Model‐X knockoff framework is the exact exchangeability of the original and knockoff variables under swapping. Matching the first two moments achieves this exact exchangeability if and only if the covariates are jointly Gaussian [18]. For non‐Gaussian covariates such as what we have, second‐order moment matching yields second‐order knockoffs which serve as a practical approximation to true exchangeability. Therefore, our use of second‐order knockoffs, while allowing us to avoid assuming making a parametric distributional assumption, provides approximate FDR control. We then calculated our knockoff feature statistics following the similar LASSO coefficient difference (LCD) statistic sets from Candès et al. [18], where we defined $Z_{j}=|{\hat{\beta }}_{j}|$ and ${\tilde{Z}}_{j}=|{\hat{\beta }}_{j+P}|$ which measure the variable importance of jth covariate, $X_{j}$ and its knockoff counterpart, ${\tilde{X}}_{j}$, respectively. The knockoff feature statistics are defined for $X_{j}$ (j=1,2,…,P) as $W_{j}=Z_{j}-{\tilde{Z}}_{j}$, and a large positive value of whom provides evidence that $X_{j}$ is important [18]. FDR control by the Model‐X knockoff procedure is guaranteed by the following theorem proposed by Candès et al. [18].

Theorem Model‐X Knockoff FDR Control.

Choose a threshold τ>0 by setting

$$\tau =\min\left\{t>0:\frac{1+\#\left\{j:W_{j}\leq -t\right\}}{\#\left\{j:W_{j}\geq t\right\}}\leq q\right\},$$

where q is the target FDRlevel. Then the procedure selecting important variables $\hat{S}=\left\{j:W_{j}\geq \tau \right\}$ controls the FDR.

Note that the construction of Model‐X knockoffs, computation of feature statistics, and determination of rejection threshold can be implemented using the existing knockoff R package [18, 33], so that users may customize their own feature statistics by defining specific functions according to needs of different statistical models instead of using the predefined test statistics. Our R code implementing our GMIFS CPM for a continuous outcome subject to DLs when the covariate space is high‐dimensional with simulation examples and application illustrations are available in our GitHub website: https://github.com/rickysunshine/CPMgmifs.

## 3 Simulations Studies

In this section, we describe our extensive simulations conducted to evaluate model performance of our proposed GMIFS CPM in high‐dimensional settings for continuous and mixed‐type outcomes with proper link function specification, including only penalized covariates (i.e., high‐throughput gene expression data as predictors). Specifically, we assessed our proposed model regarding variable selection performance with FDR control via the Model‐X knockoffs procedure under different sample sizes and effect sizes scenarios. To compare the performance of our method with that of competing approaches, we introduced four penalized linear regression models—the LASSO penalized linear regression model, the GMIFS linear regression model, the Ridge penalized linear regression model and the Elastic Net penalized linear regression model. These comparative methods were selected because they are the most frequently used methods for modeling a continuous response in high‐dimensional covariate spaces. Additionally, we included the GMIFS linear regression model because it should very closely approximate the solution path of the LASSO linear regression model [24, 25]. Further, by including the GMIFS linear regression model in the comparison of our proposed GMIFS CPM and the traditional LASSO penalized linear regression model, we are able to eliminate possible confounding effects of different penalization methods (i.e., GMIFS versus LASSO) on model performance. We note that we expect these different statistical models (i.e., the CPM versus linear regression model) will produce different performance results when applied to non‐linear continuous outcomes. We also expect the GMIFS linear regression model and the LASSO penalized linear regression model will yield nearly identical results given the GMIFS approach is a generalized extension of monotone LASSO method [14].

### 3.1 Simulation Design

We simulated high‐dimensional data in which the relationships between covariates and the outcome is known. To mimic realistic scenarios, we used gene expression data X having N×G dimensions (N≪G) from a normalized filtered RNA‐seq dataset from acute myeloid leukemia patients, from which to sample truly associated covariates. The outcome of interest Y was generated from the semiparametric linear transformation model (Equation 1), where we randomly selected p underlying truly important gene features $\left(X_{1},X_{2},\ldots ,X_{p}\right)$ out of G features to have non‐zero coefficients. Regarding these p non‐zero coefficients, half were selected to have a positive effect (i.e., β>0) and half were selected to have a negative effect (i.e., β<0), all with the same magnitude. To examine the impact of the size of covariate effects on the outcome of interest, we set the non‐zero coefficient values (β) to vary from 1 to 3 across the different simulation scenarios. All remaining βs were set to zero. In all simulations, we set p to be 20. Afterwards, we generated the observed outcome of interest $Y=H\left(Y^{*}\right)=H\left(\mathrm{X\beta}+\epsilon \right)=H\left(\beta_{1}X_{1}+\beta_{2}X_{2}+\ldots +\beta_{p}X_{p}+\epsilon \right)$.

In terms of the transformation function H(·) from the linear transformation model in (Equation 1), we chose different functions to represent different types of outcomes. For example, we used a non‐linear exponential transformation function H(t)=Exp(t) to generate continuously scaled non‐linear outcomes in our simulations. Specifically, we simulated $Y=H\left(Y^{*}\right)=\mathrm{Exp}\left(Y^{*}\right)=\mathrm{Exp}\left(\mathrm{X\beta}+\epsilon \right)=\mathrm{Exp}\left(\beta_{1}X_{1}+\beta_{2}X_{2}+\ldots +\beta_{p}X_{p}+\epsilon \right)$, where the random error term ϵ was generated from the standard normal distribution (i.e., ϵ∼N(0,1)) and the corresponding properly specified link function was probit link for our proposed CPM. Because we chose the exponential transformation function to generate non‐linearly distributed continuous outcomes which violated the linearity assumption of linear regression model, we expected that all penalized linear regression models (LASSO, GMIFS, elastic net, and ridge) would perform poorly in our non‐linear continuous outcome simulations. To mimic a realistic correlation structure observed in actual gene expression data, our simulated design matrix X used a normalized RNA‐seq expression data from AML patients, where we removed genes having all or mostly negative gene expression values, those having very low positive expression levels, and those with low variance. The final filtered data included 1154 patients and 7011 genes which served as our high‐dimensional covariate space to sample from for our simulations, $X_{\text{pool}}$. From this matrix, we randomly selected G=1500 genes and varied the number of samples (N=100,200,400,600,800,1000) to form our high‐dimensional covariate matrix X. Use of real data to construct our high‐dimensional covariate matrix instead of sampling from a multivariate Gaussian distribution allowed us to mimic the high‐dimensional gene expression data structure in real world settings, making our simulation results more informative when assessing model performance in real data applications.

Similarly, we simulated mixed‐type outcomes by setting transformation function H(·) such that it was constant on some intervals, and a continuously non‐linear exponential function elsewhere. That is

$$Y=H\left(Y^{*}\right)=\left\{\begin{array}{ll} \mathrm{Exp}\left(\tau \right) & Y^{*}\leq \tau \\ \mathrm{Exp}\left(Y^{*}\right) & \tau <Y^{*}<\psi \\ \mathrm{Exp}\left(\psi \right) & Y^{*}\geq \psi \end{array}\right.,$$

where τ is the limit of detection and ψ is the limit of quantification of latent variable $Y^{*}$, respectively. In our simulations, we choose the 10% sample quantile of $Y^{*}$ to be the limit of detection and the 85% sample quantile to be the limit of quantification to generate mixed‐type outcomes. The same generation of $Y^{*}$ and random error ϵ as continuous case were applied, where $Y^{*}=\beta_{1}X_{1}+\beta_{2}X_{2}+\ldots +\beta_{p}X_{p}+\epsilon$ and ϵ∼N(0,1) with the same properly specified probit link function for our CPM. We generated the high‐dimensional covariate space X and β as the same way as we did for continuous outcome scenario. To evaluate model performance of our proposed GMIFS CPM as thoroughly as possible, a sensitivity analysis was performed, using two different sets of sample quantiles of the latent variable $Y^{*}$ for determining the limit of detection (10% versus 20%) and the limit of quantification (85% versus 75%) for generating the mixed‐type outcomes Y. This allowed us to further examine the robustness of model performance of our proposed GMIFS CPM.

We examined multiple metrics for controlled variable selection performance evaluation and method comparison, including true positive rate (TPR), false discovery rate (FDR), $F_{1}$ score, and total discoveries. The $F_{1}$ score is a measure of predictive performance, combining precision (true discovery rate, TDR = 1—FDR) and recall (sensitivity or TPR). A high $F_{1}$ score indicates high power but a low FDR is usually desired. When the Model‐X knockoffs framework is applied so that the target FDR level is controlled under 20% in high dimensions for all methods, then the model with a higher $F_{1}$ score is preferred. Moreover, we prefer a model maintaining a high TPR but a reasonable number of total discoveries, which should be around the number of underlying true features (oracle is 20).

For both non‐linear continuous and mixed‐type outcome simulations, we examined controlled variable selection model performance under different sample sizes when we fixed the effect size at a pre‐specified value of 2 and varied the total sample size N=100,200,400,600,800,1000 for continuous outcome and N=100,200,400,500 for mixed‐type outcome, respectively. Similarly, we evaluated the impact of effect size on model performance when we fixed the total sample size N at 500 and the effect size ranged from 1 to 3 by half increment (i.e., 1,1.5,2,2.5, and 3). Additionally, we evaluated the robustness of our GMIFS CPM to link function misspecification on controlled variable selection performance for mixed‐type outcomes using the first set of sample quantiles determining the LOD (10% sample quantile) and the LOQ (85% sample quantile) across different sample sizes N=100,200,400,500 where we fixed effect size at 2. Specifically, we still generated data from the standard normal error distribution under the linear transformation model, so that the correct link function is the probit link. However, we then fit GMIFS CPM using misspecified logit and loglog link functions. Here, the logit and loglog links were chosen to represent different degrees of link function misspecification. The logit link function misspecification is moderate because it shares a similar shape and skewness to the correct probit link, whereas the loglog link function is severely misspecified due to its different shape and skewness from probit link. To better illustrate such model robustness to link function misspecification, we also included simulations results of our GMIFS CPM using the correct probit link function. For all the assessments, the simulations were replicated 100 times, and we calculated the sample means of corresponding metrics over these simulation replications to represent the average performance of each method in our simulations.

Simulations were run on the Ohio Supercomputer Center (OSC). Regarding computational times, briefly, simulations involving 10 replicates for a continuous outcome took ∼15 h—this included generating Model‐X knockoffs for 1500 penalized features and fitting our penalized CPM using GMIFS algorithm when we had a small sample size (N=100) and large effect size (i.e., signal amplitude A=3). On the other hand, simulations involving 5 replicates for mixed‐type outcome took ∼80 h when the sample size was large (N=500) and the effect size was small (A=1). All simulation scenarios involved 100 replications in total, thus we simultaneously ran 10 or 20 jobs using different random seeds to obtain 100 replicates. Therefore, each scenario took 15 h to 80 h.

### 3.2 Simulation Results

In this section, simulation results evaluating controlled variable selection performance for non‐linear continuous and mixed‐type outcomes, which were both generated by non‐linear exponential transformation function, are reported for different approaches using the metrics described in Section 3.1, including sensitivity analysis examining different levels for determining LOD and LOQ as well as for evaluating model robustness when misspecifying the link function. For both non‐linear continuous and mixed‐type outcome simulations, sample size and effect size assessment results are summarized in corresponding tables to illustrate model performance and methods comparisons. The controlled variable selection performance of each of five methods (the GMIFS CPM, the LASSO penalized linear regression model, the GMIFS linear regression model, the Elastic Net penalized linear regression model, and the Ridge penalized linear regression model) was evaluated using four metrics: TPR, FDR, $F_{1}$ score, and total discoveries and compared side‐by‐side.

#### 3.2.1 Non‐Linear Continuous Outcomes Simulation Results

The sample size assessment simulation results for non‐linear continuous outcomes regarding Model‐X knockoffs procedure controlled variable selection of our proposed GMIFS CPM, in comparison to all other penalized linear regression models, are summarized in Table 1.

**TABLE 1:** Model‐X controlled variable selection simulation results for GMIFS CPM (Column 1–4), LASSO linear regression model (Column 5–8), GMIFS linear regression model (Column 9–12), Elastic Net linear regression model (Column 13–16), and Ridge linear regression model (Column 17–20) regarding True Positive Rates (TPR), False Discovery Rates (FDR), $F_{1}$ Scores (F1), and Total Number of Discoveries (TD) by sample size (N) for non‐linear continuous outcomes when the effect size was fixed at 2, with target FDR level controlled at 20% and 20 underlying total true features.

	GMIFS CPM				LASSO linear regression model				GMIFS linear regression model				Elastic net linear regression model				Ridge linear regression model			
	TPR	FDR	F1	TD	TPR	FDR	F1	TD	TPR	FDR	F1	TD	TPR	FDR	F1	TD	TPR	FDR	F1	TD
*N* = 100	0.127	0.277	0.209	4.35	0.001	0.995	0.076	3.00	0.001	0.997	0.083	2.13	0.000	1.000	NaN	2.96	0.067	0.988	0.030	102.49
*N* = 200	0.492	0.232	0.580	13.63	0.006	0.995	0.040	6.31	0.000	1.000	NaN	0.87	0.005	0.996	0.037	6.43	0.026	0.993	0.029	34.17
*N* = 400	0.819	0.305	0.740	24.42	0.006	0.978	0.074	6.52	0.000	1.000	NaN	0.68	0.001	0.994	0.091	4.86	0.002	0.999	0.029	8.32
*N* = 600	0.925	0.303	0.782	27.74	0.010	0.956	0.076	7.18	0.002	0.943	0.095	0.70	0.010	0.889	0.080	7.31	0.003	0.963	0.091	5.08
*N* = 800	0.939	0.259	0.820	26.21	0.011	0.959	0.083	5.44	0.005	0.833	0.095	0.60	0.012	0.949	0.092	4.70	0.000	1.000	NaN	3.15
*N* = 1000	0.972	0.277	0.821	27.95	0.022	0.918	0.082	7.80	0.003	0.917	0.095	0.60	0.015	0.935	0.076	6.76	0.003	0.985	0.064	2.81

Abbreviation: NaN: Not a Number due to zeroes in both numerator and denominator for $F_{1}$ Score calculation.

From Table 1, we conclude that our proposed GMIFS CPM outperformed all other penalized linear regression models in terms of controlled variable selection performance regarding all four metrics across different sample sizes given fixed effect size. Specifically, with increasing sample sizes, our GMIFS CPM performed better and better with higher TPRs, lower FDRs and thus higher $F_{1}$ scores. The total numbers of discoveries identified by our GMIFS CPM increased and were just slightly higher than total number of underlying true features 20. The FDRs for our method were generally around the target FDR level of 20% with the highest value of 30%. On the other hand, the competing methods—the LASSO linear regression model, the GMIFS linear regression model, Elastic Net linear regression model and the Ridge linear regression model, performed similarly and worse than our GMIFS CPM, with much lower TPRs and $F_{1}$ scores as well as much higher FDRs, with fewer total number of discoveries.

Similarly, the effect size assessment simulation results for non‐linear continuous outcomes using the Model‐X knockoffs procedure controlled variable selection of our proposed GMIFS CPM, in comparison to all other penalized linear regression models are summarized in Table 2.

**TABLE 2:** Model‐X controlled variable selection simulation results for GMIFS CPM (Column 1–4), LASSO linear regression model (Column 5–8), GMIFS linear regression model (Column 9–12), Elastic Net linear regression model (Column 13–16), and Ridge linear regression model (Column 17–20) regarding True Positive Rates (TPR), False Discovery Rates (FDR), $F_{1}$ Scores (F1), and Total Number of Discoveries (TD) by effect size for non‐linear continuous outcomes when the sample size was fixed at 500, with target FDR level controlled at 20% and 20 underlying total true features.

	GMIFS CPM				LASSO linear regression model				GMIFS linear regression model				Elastic net linear regression model				Ridge linear regression model			
	TPR	FDR	F1	TD	TPR	FDR	F1	TD	TPR	FDR	F1	TD	TPR	FDR	F1	TD	TPR	FDR	F1	TD
Effect size = 1	0.813	0.222	0.783	21.67	0.007	0.984	0.063	5.22	0.005	0.865	0.095	0.74	0.008	0.976	0.069	5.14	0.001	0.998	0.052	6.01
Effect size = 1.5	0.861	0.260	0.785	24.14	0.010	0.978	0.063	6.50	0.005	0.886	0.095	0.88	0.007	0.986	0.060	6.32	0.000	1.000	NaN	6.36
Effect size = 2	0.884	0.266	0.790	25.33	0.013	0.964	0.067	6.06	0.005	0.884	0.095	0.86	0.009	0.976	0.066	5.78	0.002	0.997	0.086	6.48
Effect size = 2.5	0.890	0.244	0.806	24.46	0.014	0.962	0.066	5.89	0.005	0.880	0.095	0.83	0.006	0.991	0.060	5.53	0.000	1.000	NaN	6.07
Effect size = 3	0.902	0.258	0.805	25.19	0.016	0.960	0.066	6.23	0.005	0.884	0.095	0.86	0.007	0.987	0.061	5.66	0.000	1.000	NaN	6.11

Abbreviation: NaN: Not a umber due to zeroes in both numerator and denominator for $F_{1}$ Score calculation.

From Table 2, we conclude that different effect sizes will not affect controlled variable selection performance of our GMIFS CPM too much given the sample size is large enough (e.g., N=500 here). In comparison to all penalized linear regression models, our GMIFS CPM always outperformed in terms of much higher TPRs, lower FDRs, and thus higher $F_{1}$ Scores. Similar to the previous sample size assessment, the FDRs of our method were slightly higher than target FDR level 20% but lower than 30% level, which indicates reasonable control of FDR based on our GMIFS CPM. Additionally, our GMIFS CPM identified a reasonable number of discoveries, which were slightly higher than the total number of underlying true features, 20, whereas the penalized linear regression models had too few discoveries. Following the sample size assessment, the LASSO linear regression model and the GMIFS linear regression model performed similarly and worse than our GMIFS CPM across all effect sizes when sample size was large (N=500).

Therefore, based on both sample size and effect size simulation results for non‐linear continuously scaled outcomes, we conclude that our GMIFS CPM performed better than penalized linear regression models regarding controlled variable selection performance when such continuous outcomes were generated by a non‐linear transformation function. This is reasonable and perhaps expected because the penalized linear regression models assume normality and linearity but our non‐linearly distributed outcomes violate these model assumptions, which in turn corresponds to poor model performance even for continuously scaled outcomes. On the other hand, our GMIFS CPM does not assume a specific form of the transformation function and underlying distribution on the outcome data, thus it works well with any type of outcome and should be preferred over traditional penalized linear regression models when the outcome data are believed to follow non‐linear continuous distributions in high dimensions regarding variable selection.

#### 3.2.2 Mixed‐Type Outcomes Simulation Results

Following a procedure similar to the continuously scaled outcome simulation scenarios, we also evaluated controlled variable selection model performance of our GMIFS CPM, in comparison to penalized linear regression models, when the outcome data are mixed‐type. As mentioned in Section 3.1, two different sets of sample quantiles of the latent variable $Y^{*}$ were used when determining the LOD and the limit of quantification, to generate mixed‐type outcomes of interest for sensitivity analysis. Specifically, the first simulation scenario for mixed‐type outcomes used the 10% sample quantile as the LOD and the 85% sample quantile as the limit of quantification, while the second simulation scenario for mixed‐type outcomes used the 20% sample quantile as the LOD and the 75% sample quantile as the limit of quantification. Because a non‐linear exponential transformation function $Y=\mathrm{Exp}\left(Y^{*}\right)$ was chosen, the actual limit of detection (${\mathrm{LOD}}^{*}$) and the actual limit of quantification (${\mathrm{LOQ}}^{*}$) for determining the left and right‐censored observed outcomes, Y for mixed‐type scale generation were in an exponential transformed scale (i.e., ${\mathrm{LOD}}^{*}=\mathrm{Exp}\left(\tau \right)$ and ${\mathrm{LOQ}}^{*}=\mathrm{Exp}\left(\psi \right)$, where τ was the LOD and ψ was the limit of quantification of latent variable $Y^{*}$, respectively). To simplify the notation, the first scenario is referred to as Scenario 1 and the other as Scenario 2 in the subsequent discussion.

##### 3.2.2.1 Scenario 1

The simulations evaluating the impact of sample size on mixed‐type outcomes when the 10% sample quantile was used as the LOD and the 85% sample quantile was used as the LOQ together with Model‐X knockoffs controlled variable selection procedure for our proposed GMIFS CPM in comparison to penalized linear regression models are summarized in Table 3.

**TABLE 3:** Model‐X controlled variable selection simulation results for GMIFS CPM (Column 1–4), LASSO linear regression model (Column 5–8), GMIFS linear regression model (Column 9–12), Elastic Net linear regression model (Column 13–16), and Ridge linear regression model (Column 17–20) regarding True Positive Rates (TPR), False Discovery Rates (FDR), $F_{1}$ cores (F1), and Total Number of Discoveries (TD) by sample size (N) for ixed‐type outcomes using the 10% sample quantile as the LOD and the 85% sample quantile as the LOQ when the effect size was fixed at 2, with target FDR level controlled at 20% and 20 underlying total true features.

	GMIFS CPM				LASSO linear regression model				GMIFS linear regression model				Elastic net linear regression model				Ridge linear regression model			
	TPR	FDR	F1	TD	TPR	FDR	F1	TD	TPR	FDR	F1	TD	TPR	FDR	F1	TD	TPR	FDR	F1	TD
*N* = 100	0.092	0.323	0.173	4.09	0.007	0.823	0.092	0.91	0.010	0.730	0.095	0.75	0.005	0.852	0.093	0.77	0.274	0.981	0.041	263.84
*N* = 200	0.372	0.258	0.482	10.40	0.012	0.700	0.115	0.81	0.033	0.257	0.109	1.03	0.009	0.754	0.118	0.77	0.204	0.984	0.034	238.62
*N* = 400	0.726	0.204	0.742	19.06	0.091	0.373	0.223	3.44	0.048	0.334	0.155	1.29	0.095	0.319	0.218	3.50	0.058	0.980	0.038	60.38
*N* = 500	0.817	0.248	0.763	23.00	0.140	0.285	0.271	4.46	0.024	0.427	0.127	0.75	0.127	0.285	0.254	4.19	0.027	0.961	0.056	22.72

From Table 3, we conclude that our GMIFS CPM outperformed all four penalized linear regression models in terms of all four metrics evaluating controlled variable selection performance when the outcomes are of mixed‐type. This conclusion is similar to our non‐linear continuously scaled outcome results. Specifically, as the sample size increased given a fixed effect size at 2, our GMIFS CPM performed better with higher TPRs, lower FDRs, and thus higher $F_{1}$ Scores. It is worth noting that the controlled variable selection performance of our GMIFS CPM did not improve too much when the sample size was larger than 400. The FDRs of our GMIFS CPM were higher at the beginning for the smallest sample size (N=100), but decreased and became flat around target FDR level 20% when sample size increased. This indicates that our GMIFS CPM can control the FDR well under the Model‐X knockoff procedure when sample size is fairly large (i.e., over 400 here). The total number of discoveries identified by our GMIFS CPM increased when sample size increased and were around the total number of true underlying features, 20. On the other hand, all penalized linear regression models performed poor having much lower TPRs, higher and unstable FDRs, much lower $F_{1}$ Scores, and much fewer total discoveries. Taken together, this indicates that penalized linear regression models should not be considered for controlled variable selection inference when the outcome data are of mixed‐type.

The simulations evaluating the impact of effect size on mixed‐type outcomes when the 10% sample quantile was used as the LOD and the 85% sample quantile was used as the LOQ together with Model‐X knockoffs controlled variable selection procedure for our proposed GMIFS CPM in comparison to penalized linear regression models are summarized in Table 4.

**TABLE 4:** Model‐X controlled variable selection simulation results for GMIFS CPM (Column 1–4), LASSO linear regression model (Column 5–8), GMIFS linear regression model (Column 9–12), Elastic Net linear regression model (Column 13–16), and Ridge linear regression model (Column 17–20) regarding True ositive ates (TPR), alse iscovery ates (FDR), $F_{1}$ cores (F1), and otal umber of iscoveries (TD) by effect size for ixed‐type utcomes using the 10% sample quantile as the LOD and the 85% sample quantile as the LOQ when the sample size was fixed at 500, with target FDR level controlled at 20% and 20 underlying total true features.

	GMIFS CPM				LASSO linear regression model				GMIFS linear regression model				Elastic net linear regression model				Ridge linear regression model			
	TPR	FDR	F1	TD	TPR	FDR	F1	TD	TPR	FDR	F1	TD	TPR	FDR	F1	TD	TPR	FDR	F1	TD
Effect size = 1	0.748	0.171	0.760	18.95	0.182	0.285	0.311	5.38	0.041	0.433	0.195	1.18	0.168	0.265	0.306	4.68	0.033	0.968	0.045	35.37
Effect size = 1.5	0.802	0.217	0.772	21.26	0.148	0.298	0.289	4.60	0.022	0.348	0.113	0.66	0.132	0.278	0.256	4.07	0.035	0.965	0.057	23.74
Effect size = 2	0.793	0.220	0.765	21.48	0.141	0.282	0.283	4.43	0.027	0.446	0.126	0.89	0.125	0.266	0.255	3.98	0.032	0.969	0.057	21.68
Effect size = 2.5	0.821	0.221	0.781	21.98	0.135	0.328	0.285	4.18	0.032	0.251	0.118	0.83	0.121	0.330	0.243	3.82	0.020	0.972	0.052	20.09
Effect size = 3	0.818	0.218	0.780	21.95	0.114	0.279	0.254	3.53	0.033	0.343	0.114	0.96	0.102	0.292	0.233	3.25	0.025	0.966	0.064	17.90

From Table 4, we conclude that different effect sizes have little affect on controlled variable selection performance of our GMIFS CPM when there is a large sample size (i.e., the sample size was fixed at 500). This finding is consistent with previous results and conclusions of our effect size assessment on non‐linear continuous outcomes. Similar to the sample size assessment for mixed‐type outcomes, our GMIFS CPM always performed much better than all four penalized linear regression models by identifying more truly important features (higher TPRs) while making fewer false discoveries (lower FDRs) across all effect size scenarios. Neither the LASSO linear regression model nor the GMIFS linear regression model performed well regarding controlled variable selection for mixed‐type outcomes, and their poor performance was similar to some extent.

Therefore, according to both sample size and effect size assessments of our simulation results for mixed‐type outcomes, we conclude that our GMIFS CPM combined with the Model‐X knockoffs procedure outperformed all penalized linear regression models with respect to controlled variable selection performance. These results are expected because such non‐linearly distributed outcomes with mixed‐type structures further violates the underlying linear regression model assumptions of penalized linear regression models, resulting in poor model fit and the inability to perform controlled variable selection inference. However, our proposed GMIFS CPM depends neither on assumptions on specific forms of the transformation function nor on the underlying distribution of outcomes, thus it is flexible with any type of outcome data and should be preferred over penalized linear regression models when outcomes of interest are of mixed‐type in high dimensions for controlled variable selection inference.

##### 3.2.2.2 Scenario 2

To evaluate robustness of our proposed GMIFS CPM with respect to different thresholds determining LOD and LOQ for mixed‐type outcomes generation on controlled variable selection performance, a sensitivity analysis was conducted by generating the mixed‐type outcomes using the 20% sample quantile as the LOD and the 75% sample quantile as the LOQ. All simulation procedures and settings were the same as Scenario 1, except for using different thresholds for specifying the LOD and LOQ for mixed‐type outcome data generation.

The sample size assessment simulation results for mixed‐type outcomes when the 20% sample quantile was used as the LOD and the 75% sample quantile was used as the LOQ together with Model‐X knockoffs controlled variable selection procedure for our proposed GMIFS CPM in comparison to all penalized linear regression models are summarized in Table 5.

**TABLE 5:** Model‐X controlled variable selection simulation results for GMIFS CPM (Column 1–4), LASSO linear regression model (Column 5–8), GMIFS linear regression model (Column 9–12), Elastic Net linear regression model (Column 13–16), and Ridge linear regression model (Column 17–20) regarding true Positive ates (TPR), alse iscovery ates (FDR), $F_{1}$ cores (F1), and otal umber of iscoveries (TD) by sample size (N) for ixed‐type utcomes using the 20% sample quantile as the LOD and the 75% sample quantile as the LOQ when the effect size was fixed at 2, with target FDR level controlled at 20% and 20 underlying total true features.

	GMIFS CPM				LASSO linear regression model				GMIFS linear regression model				Elastic net linear regression model				Ridge linear regression model			
	TPR	FDR	F1	TD	TPR	FDR	F1	TD	TPR	FDR	F1	TD	TPR	FDR	F1	TD	TPR	FDR	F1	TD
*N* = 100	0.043	0.355	0.145	1.39	0.002	0.950	0.085	0.60	0.017	0.528	0.097	0.71	0.000	1.000	NaN	0.39	0.319	0.978	0.040	367.56
*N* = 200	0.245	0.183	0.368	6.54	0.031	0.557	0.128	1.42	0.047	0.324	0.160	1.49	0.024	0.673	0.122	1.40	0.188	0.985	0.029	257.49
*N* = 400	0.636	0.215	0.665	17.60	0.122	0.224	0.231	3.87	0.048	0.262	0.143	1.26	0.097	0.278	0.204	3.28	0.092	0.972	0.033	135.32
*N* = 500	0.737	0.176	0.745	18.77	0.183	0.224	0.287	5.12	0.019	0.383	0.104	0.59	0.178	0.277	0.291	5.12	0.056	0.977	0.072	39.13

Abbreviation: NaN: Not a umber due to zeroes in both numerator and denominator for $F_{1}$ core calculation.

From Table 5, our conclusion follows that from Scenario 1, that our GMIFS CPM outperformed all penalized linear regression models regarding four metrics evaluating controlled variable selection performance across different sample sizes, given a fixed effect size at 2, for mixed‐type outcomes. Specifically, as the sample size increased with the effect size fixed, our method selected more truly important variables while making fewer false discoveries such that the overall selection performance measured by $F_{1}$ scores improved. The FDR control of our GMIFS CPM was good because most of FDRs were around the target FDR level 20%, except for the first entry when sample size N=100, which was expected because small sample size will undermine the controlled variable selection performance of our method.

The effect size assessment simulation results for mixed‐type outcomes when the 20% sample quantile was used as the LOD and the 75% sample quantile was used as the LOQ together with Model‐X knockoffs controlled variable selection procedure for our proposed GMIFS CPM are summarized in Table 6.

**TABLE 6:** Model‐X controlled variable selection simulation results for GMIFS CPM (Column 1–4), LASSO linear regression model (Column 5–8), GMIFS linear regression model (Column 9–12), Elastic Net linear regression model (Column 13–16), and Ridge linear regression model (Column 17–20) regarding true positive rates (TPR), false discovery rates (FDR), $F_{1}$ cores (F1), and otal umber of iscoveries (TD) by effect size for ixed‐type utcomes using the 20% sample quantile as the LOD and the 75% sample quantile as the LOQ when the sample size was fixed at 500, with target FDR level controlled at 20% and 20 underlying total true features.

	GMIFS CPM				LASSO linear regression model				GMIFS linear regression model				Elastic net linear regression model				Ridge linear regression model			
	TPR	FDR	F1	TD	TPR	FDR	F1	TD	TPR	FDR	F1	TD	TPR	FDR	F1	TD	TPR	FDR	F1	TD
Effect size = 1	0.678	0.134	0.727	16.37	0.206	0.201	0.343	5.55	0.087	0.102	0.283	2.10	0.182	0.186	0.319	4.77	0.023	0.987	0.041	39.02
Effect size = 1.5	0.766	0.168	0.764	19.45	0.236	0.236	0.358	6.80	0.036	0.391	0.141	1.53	0.229	0.291	0.351	7.05	0.056	0.974	0.060	50.99
Effect size = 2	0.784	0.200	0.758	21.01	0.229	0.256	0.358	6.42	0.029	0.434	0.133	1.26	0.215	0.268	0.355	5.89	0.051	0.979	0.068	40.51
Effect size = 2.5	0.790	0.197	0.767	20.77	0.203	0.231	0.316	5.54	0.013	0.500	0.095	0.51	0.182	0.256	0.317	5.10	0.028	0.980	0.065	24.46
Effect size = 3	0.799	0.192	0.773	20.80	0.196	0.233	0.311	5.39	0.016	0.446	0.095	0.56	0.172	0.250	0.290	4.86	0.022	0.981	0.063	22.15

From Table 6, we again conclude that effect size has little affect on controlled variable selection performance of our GMIFS CPM, which is consistent with our results from Scenario 1 and the non‐linear continuous case. Similar to the sample size assessment results, our GMIFS CPM always performed better than all penalized linear regression models in terms of higher TPRs and higher $F_{1}$ Scores across all effect sizes when the sample size was fairly large. The FDRs of our method were controlled well because values were either below or at target FDR level 20% with different effect sizes. The total number of discoveries identified by our GMIFS CPM increased at first, but then stayed close to the total number of true underlying features, 20, indicating that our method can efficiently identify important variables without making too many false discoveries. On the other hand, however, neither the LASSO linear regression model nor the GMIFS linear regression model performed well regarding identifying more important features while avoiding too many false discoveries. Fewer total number of discoveries from these penalized linear regression models also illustrates the poor controlled variable selection performance when outcome data are of mixed‐type. Therefore, considering both sample size and effect size assessment simulation results in Scenario 2, we reach the same conclusion as in Scenario 1, that our GMIFS CPM outperformed competing penalized linear regression models with respect to controlled variable selection performance when the outcome data are of mixed‐type.

Comparing Scenario 1 with Scenario 2 using two different sets of thresholds as LOD and LOQ for mixed‐type data generation, we conclude that our GMIFS CPM was robust to different choices of LOD and LOQ regarding controlled variable selection performance because simulation results and corresponding conclusions remained the same. Our GMIFS CPM always performed better than the other four penalized linear regression models with different sample sizes and effect sizes in terms of controlled variable selection performance for mixed‐type outcomes in high dimensions.

#### 3.2.3 Evaluation of Robustness to Link Function Misspecification

**FIGURE A1:**
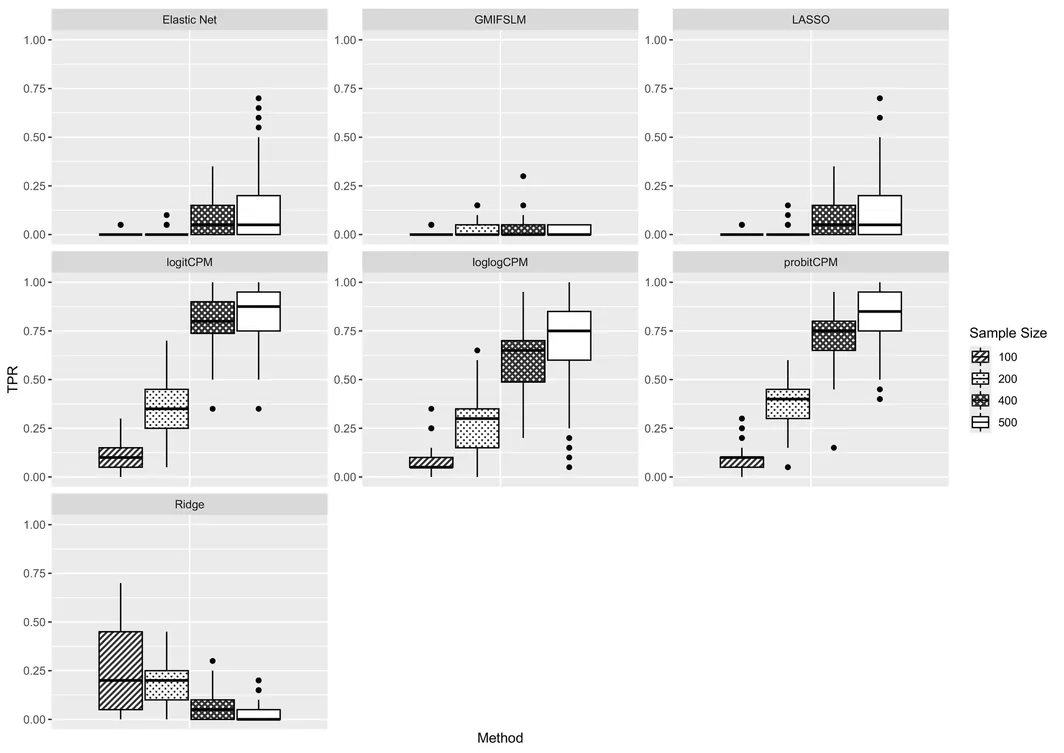
Boxplots of True Positive Rates (TPR) for different methods for modeling a mixed‐type outcome when the sample size is varied as N=100,200,400,500 and the effect size is fixed at 2.

**FIGURE A2:**
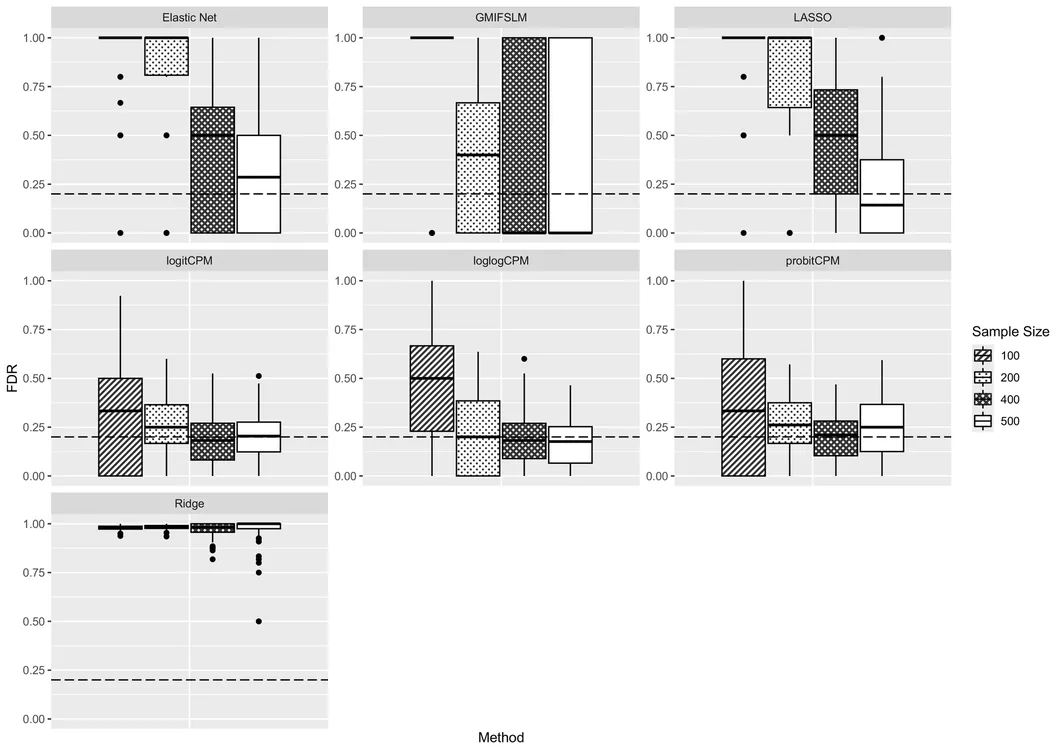
Boxplots of False Discovery Rates (FDR) for different methods for modeling a mixed‐type outcome when the sample size is varied as N=100,200,400,500 and the effect size is fixed at 2. Note that the dash line represents the 20% target FDR level.

**FIGURE A3:**
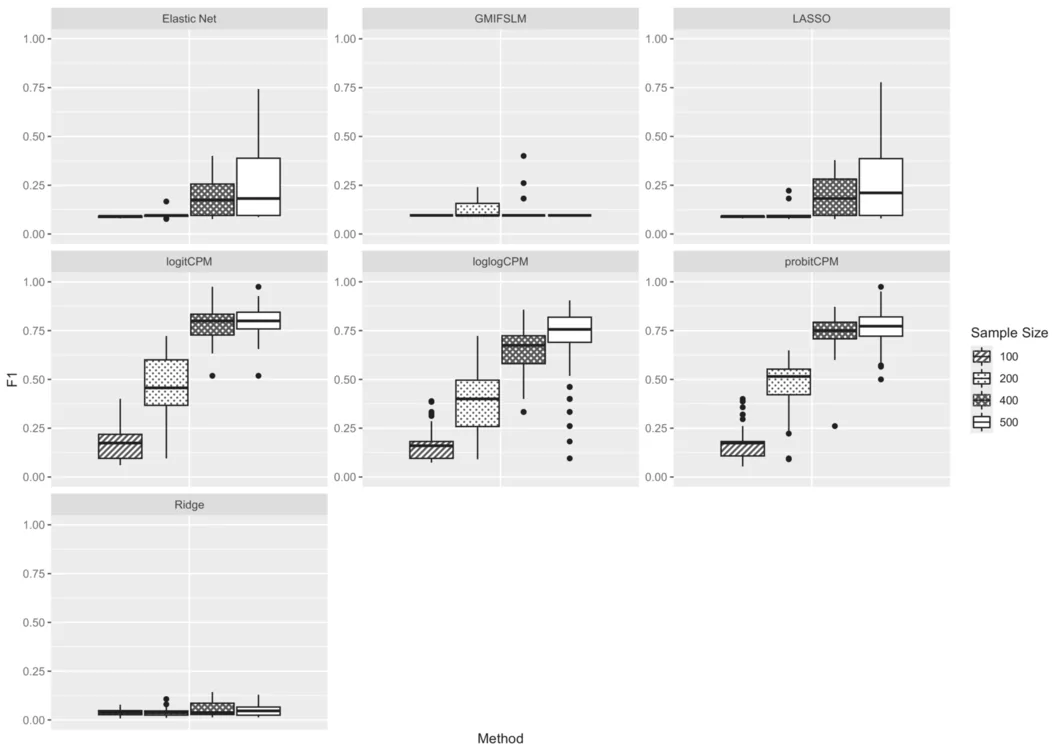
Boxplots of F1 Scores for different methods for modeling a mixed‐type outcome when the sample size is varied as N=100,200,400,500 and the effect size is fixed at 2.

When evaluating the robustness to link function misspecification, we additionally included elastic net and ridge penalized linear regression models. The simulation results are summarized via boxplots of TPR, FDR and $F_{1}$ scores (Figures A1, A2, and A3 in the Appendix, respectively) over all simulation replications for each of seven methods compared, including our GMIFS CPM with probit, logit or loglog link, LASSO penalized linear regression model, GMIFS linear regression model, Elastic Net penalized linear regression model and Ridge penalized linear regression model. From these simulation results, we conclude that our proposed GMIFS CPM consistently outperformed the traditional penalized linear regression models (LASSO, elastic net, ridge) even when the fitted GMIFS CPM link function was misspecified, with respect to controlled variable selection. Further, our GMIFS CPM approach's performance improved as the sample size increased. When our GMIFS CPM was minimally misspecified using logit link, controlled variable selection performance was quite similar to that from the correct probit link model. This is perhaps expected because the logit and probit links share similar shapes and skewness. However, when the GMIFS CPM was moderately to severely misspecified using the log−log link, controlled variable selection performance of our proposed CPM was negatively impacted to some extent across all sample sizes. Nevertheless, the misspecified link GMIFS CPM still well‐outperformed all other penalized linear regression models across all sample sizes. Therefore, we conclude that our GMIFS CPM is robust to link function misspecification for controlled variable selection for mixed‐type outcomes, and that a larger sample size improves variable selection performance of our penalized CPM in high‐dimensions.

Finally, given the simulation results from both non‐linear continuously scaled and mixed‐type outcomes, our proposed GMIFS CPM can identify important variables while avoiding too many false discoveries among hundreds and thousands candidates in high‐dimensional genomic settings when the outcome data is non‐linear. Specifically, when the outcomes of interest are believed to follow some non‐linear distribution or to have LOD and/or limit of quantification, our GMIFS CPM should be preferred over traditional penalized linear regression methods for controlled variable selection inference in high‐dimensions. Additionally, we found that there was no obvious difference in model performance between the LASSO linear regression model and the GMIFS linear regression model, providing evidence that it is the different statistical model (CPM versus linear regression model) rather than the penalization procedure that yielded the different performance results. The robustness property of our GMIFS CPM to link function misspecification provides flexibility of link selection in practice, where we may still fit a reasonably appropriate GMIFS CPM model to the data for valid variable selection even if we do not know the underlying true error distribution in the real‐world application.

## 4 Application

The motivation behind our proposed GMIFS CPM derives from the need to model a continuous outcome subject to a clinical reporting threshold. In this section, we analyzed a real dataset to demonstrate clinical application of our GMIFS CPM in a real‐world setting. Specifically, we illustrate the application of our GMIFS CPM for a mixed‐type outcome, estimated glomeruli filtration rate (eGFR), by performing controlled variable selection using a high‐dimensional gene expression dataset from kidney transplant recipients.

### 4.1 Data

We applied our proposed GMIFS CPM to the kidney transplant recipient dataset that included gene expression measured using Affymetrix GeneChip microarrays (HG‐U133A 2.0) from 174 deceased donor kidneys biopsies that were subsequently implanted into the recipients (access: GSE147451). After filtering to remove probe sets absent in all samples and control probe sets, there were 19 369 probe sets for the 174 samples. The 174 kidney transplant recipients were then followed and their corresponding eGFRs were measured at 24 months post‐transplant using the abbreviated Modification of Diet in Renal Disease (MDRD) formula [2, 34]. As introduced in the previous Section 1, eGFR, the outcome of interest, is of mixed‐type, which is partially continuous for values below 60, and then reaches a clinical reporting threshold when values are above 60, such that eGFR values ≥60 represent unquantifiable good kidney functioning.

The original gene expression dataset included 19 369 probe sets after initial filtering. However, it is often the case that there exists a portion of gene features that are considered to be “noise” because of their trivial expression values and/or low variabilities, making them less likely to be the candidates or important variables associated with the outcome of interest. Therefore, gene expression data are usually pre‐processed or screened to further remove those “noise” variables and retain only the genes expressed at reliably measured levels as candidates to form the high‐dimensional covariate space for subsequent modeling. With respect to screening methods, the most common method is to filter out variables having relatively low expression values and/or variabilities according to some pre‐specified thresholds. In this analysis, we followed similar screening process used by the original authors [2], and fit probe set level ordinal CPMs to the mixed‐type outcome eGFR. Keeping probe sets as those most expressed, defined by those exceeding the first sample quartile (Q1), and then applying controlled FDR using Benjamini and Hochberg's procedure [35] to retain probe sets significant at an FDR <0.20, this procedure identified 567 probe sets to retain for multivariable modeling. Thereafter, the probe set data matrix was filtered to retain these 567 probe sets, resulting in a final filtered gene expression dataset as our high‐dimensional covariate space that included 174 subjects with 567 probe sets. Thus, Model‐X knockoffs were derived for this pre‐filtered dataset.

### 4.2 Gene Discovery

A GMIFS CPM was fit to the mixed‐type eGFR at 24 months post‐transplant where 567 probe sets comprised the penalized covariate space, while controlling the FDR at 5% via Model‐X knockoffs procedure [18], to identify important genes associated with mixed‐type eGFR outcome simultaneously.

Table 7 shows the identified five gene symbols (*HDLBP*, *SMARCC2*, *GYPC*, *ZNF185*, and *KPNB1*) corresponding to probe sets associated with mixed‐type eGFR of our GMIFS CPM with respect to estimated coefficients $\left(\hat{\beta }\right)$, computed Model‐X knockoffs feature statistics (W) and rejection threshold (τ) with control of FDR ≤5%. In comparison to our method, both the LASSO linear regression model and the GMIFS linear regression model were fit to the same kidney transplantation study data with an FDR at the same level, 5%, where these two penalized linear regression models identified the same single gene feature (*ZNF185*). Because three methods all selected this *ZNF185* gene feature, it may suggest that this gene may be highly associated with eGFR and thus could be a strong prognostic factor of kidney functional outcomes.

**TABLE 7:** List of GMIFS CPM identified genes associated with eGFR in kidney transplant recipients 24 months post‐transplant among 567 candidates, controlling FDR ≤5%.

Gene symbol	$\hat{\beta }$	W	τ
*HDLBP*	0.293	0.293	0.271
*SMARCC2*	0.387	0.387	
*GYPC*	−0.271	0.271	
*ZNF185*	0.289	0.289	
*KPNB1*	0.336	0.336	

*Note:* The$\hat{\beta }$ represents corresponding estimated coefficients of identified genes, W represents Model‐X knockoffs feature statistics and τ is the data‐dependent rejection threshold while controlling the target FDR ≤5%.

### 4.3 Validation of the Identified Genes

To validate gene discovery performance of three methods to some extent, we examined the biological importance of our GMIFS CPM, the LASSO linear regression model and the GMIFS linear regression model identified gene features by searching whether any of the genes included in the models are linked to renal disease in PubMed and/or The Human Protein Atlas.

From Table 8, there are 4 out of 5 (80%) our GMIFS CPM identified genes that can be linked to kidney disease (particularly renal cancers), including *HDLBP*, *SMARCC2*, *ZNF185*, and *KPNB1*. This result is reasonable because renal cancer is often the worsening consequence of kidney disease and biologically genes associated with inflammation are also often linked to renal cancers. On the other hand, both the LASSO linear regression model and the GMIFS linear regression model only identified single gene feature, *ZNF185*, which has been validated for its biological importance to renal disease—KIRC, which is a main histological subtype of RCC [36] in our method. In comparison to competing penalized linear regression models, our GMIFS CPM identified five important genes associated with mixed‐type outcome eGFR among 567 candidates, and four of them were linked to kidney disease, validating their biological importance. If we focus on GMIFS CPM exclusively identified genes, there were 3 out of 4 (75%) genes having biological importance of kidney disease, whereas neither the LASSO linear regression model nor the GMIFS linear regression model exclusively identified any gene.

**TABLE 8:** List of biological importance validation of gene discovery by GMIFS CPM. Note that “RCC” represents renal cell carcinoma, “KIRC” represents kidney renal clear cell carcinoma, “renal cancer” means that it is either pathogenic factor to kidney tumors for HDLBP or known prognostic marker in renal cancers for KPNB1 and “NA” means that there is no biological importance information associated with renal disease available for that gene feature.

Gene symbol	Kidney disease
*HDLBP*	Renal cancer
*SMARCC2*	RCC
*ZNF185*	KIRC
*KPNB1*	Renal cancer
*GYPC*	NA

In conclusion, our GMIFS CPM can identify important gene features that are highly related to mixed‐type kidney functional outcome (e.g., eGFR) in a high‐dimensional genomic setting, while controlling FDR at target level in this kidney transplantation application. The LASSO linear regression model and the GMIFS linear regression model showed the same poor controlled features identification performance in comparison to our method. This is expected because our simulations results have shown that two penalized linear regression models are not appropriate for high‐dimensional controlled variable selection when outcome of interest is of mixed‐type. Therefore, our GMIFS CPM should be preferred over other penalized methods when modeling a mixed‐type outcome using high‐dimensional covariate space regarding controlled features identification. It can select succinct lists of important genes which are biologically related to disease outcome of interest, which may further in turn provide prognostic guidance in a real‐world setting.

## 5 Discussion and Conclusion

In this article, we proposed the GMIFS CPM to model a mixed‐type outcome for high‐dimensional data. We extended the CPM for mixed‐type outcome data arising from a continuous outcome that may be subject to detection limits by penalizing high‐throughput gene expression features via the GMIFS algorithm, to identify important biomarkers associated with outcomes of interest. The estimation algorithm of our proposed method has been further combined with the Model‐X knockoffs framework for FDR control. Results from our extensive simulation studies demonstrate that our proposed GMIFS CPM outperformed competing penalized linear regression models in terms of controlled variable selection in high dimensions for both non‐linear continuous and mixed‐type outcome data. Specifically, our method achieved high power with the FDR being controlled at the target level after adopting the Model‐X knockoff framework. Moreover, our simulation studies revealed that it is the statistical model, rather than the penalization method, that yielded different performance results. From robustness analysis, we conclude that our GMIFS CPM is robust to link function misspecification on controlled variable selection inference, which eases the dependency on the choice of link function in practice. In the kidney transplant application, important genes identified to be associated with the mixed‐type outcome, eGFR, may provide informative implications for renal disease research or clinical practice paradigms.

One shortcoming of our study is that we did not consider coercing selected covariates into the model. That is, perhaps some demographic or clinical characteristics (e.g., age, sex) should be coerced into the model (unpenalized). Including unpenalized predictors into our model may be effective in removing confounding effects associated with known demographic and/or clinical characteristics. A key methodological choice in our optimization framework is fixing the α thresholds at the initial step rather than re‐estimating them continuously throughout the iterative procedure. While simultaneously optimizing α and β is theoretically ideal, it introduces an extreme computational burden when modeling continuous outcomes, where the dimensionality of α scales with the sample size. Fixing α based on the empirical baseline distribution of the outcome represents a pragmatic trade‐off. Theoretically, this two‐step approach could introduce a slight estimation bias if the true conditional distribution drastically violates the baseline structural assumptions. However, from a variable selection perspective, freezing the thresholds stabilizes the parameter space and eliminates the variance inflation that typically occurs when intercepts and coefficients shift concurrently. Our strong empirical results suggest that this strategy yields a robust, computationally scalable approach to variable selection without sacrificing predictive accuracy, though future work could explore standard error corrections or localized threshold tuning. A third limitation is that simulation studies were performed limiting the number of features to P=1,500. While this reduced the computational burden allowing us to vary sample size and effect size, it does not fully evaluate performance when fitting models to large unfiltered genomic datasets. Finally, in our application, we confined Model‐X knockoff generation to the 567 pre‐filtered probe sets because their generation to the full dataset of 19 369 probe sets is too computationally demanding. Our results are therefore conditional to pre‐filtering of the data.

In the future, we are interested in developing a cross‐validation procedure for tuning and selecting an optimal step of our GMIFS CPM. We also plan to provide an unpenalized option to increase flexibility of our model. Aside from non‐linear and mixed‐type outcomes, our model may also be appropriate for modeling a discrete ordinal response variable having a large number of levels (e.g., FIGO stage from cervical cancer has up to 17 ordinal levels) in a high‐dimensional genomic setting. Existing penalized ordinal regression models may fail to model such ordinal response due to the large number of ordinal levels.

## Funding

This research was supported by the National Institute of Diabetes and Digestive and Kidney Diseases (NIDDK) of the National Institutes of Health under awards numbers: R01DK109581 (V.R.M./K.J.A.) and R21AI172077 (V.R.M./K.J.A.).

## Disclosure

The authors have nothing to report.

## Conflicts of Interest

The authors declare no conflicts of interest.

## Data Availability

The data that support the findings of this study are openly available in Gene Expression Omnibus at http://www.ncbi.nlm.nih.gov/geo/query, reference number GSE147451. R code that implement the generalized monotone incremental forward stagewise algorithm for fitting cumulative probability models is available at GitHub.com/rickysunshine/CPMgmifs.
